# Application of Gaussian SVM Flame Detection Model Based on Color and Gradient Features in Engine Test Plume Images

**DOI:** 10.3390/s25175592

**Published:** 2025-09-08

**Authors:** Song Yan, Yushan Gao, Zhiwei Zhang, Yi Li

**Affiliations:** 1 National Key Laboratory of Aerospace Liquid Propulsion, Xi’an Aerospace Propulsion Institute, Xi’an 710100, China; ys060599@126.com (S.Y.); qinghuazhiwei@163.com (Z.Z.); lybyy289@163.com (Y.L.); 2 Academy of Aerospace Propulsion Technology, Xi’an 710100, China

**Keywords:** liquid rocket engine, flame detection, color features, gradient features, support vector machine (SVM), exhaust plume

## Abstract

This study presents a flame detection model that is based on real experimental data that were collected during turbopump hot-fire tests of a liquid rocket engine. In these tests, a MEMRECAM ACS-1 M40 high-speed camera—serving as an optical sensor within the test instrumentation system—captured plume images for analysis. To detect abnormal flame phenomena in the plume, a Gaussian support vector machine (SVM) model was developed using image features that were derived from both color and gradient information. Six representative frames containing visible flames were selected from a single test failure video. These images were segmented in the YCbCr color space using the *k*-means clustering algorithm to distinguish flame and non-flame pixels. A 10-dimensional feature vector was constructed for each pixel and then reduced to five dimensions using the Maximum Relevance Minimum Redundancy (mRMR) method. The reduced vectors were used to train the Gaussian SVM model. The model achieved a 97.6% detection accuracy despite being trained on a limited dataset. It has been successfully applied in multiple subsequent engine tests, and it has proven effective in detecting ablation-related anomalies. By combining real-world sensor data acquisition with intelligent image-based analysis, this work enhances the monitoring capabilities in rocket engine development.

## 1. Introduction

The turbopump joint test and subsystem test of liquid rocket engines are crucial for evaluating system compatibility and reliability during engine development. During these tests, parameters such as the rotational speed, temperature, and pressure of the engine’s turbopump are monitored. However, these parameters are not sensitive to certain fault modes (e.g., localized ablation) and often show delays, making it difficult to accurately pinpoint the onset of faults and, thus, complicating fault cause analysis. Plume images can reflect the internal operating conditions of the engine; minor rubbing or localized ablation faults, for instance, can cause changes in the plume color. These color variations can be used for fault identification and localization. In a typical turbopump joint test, the nozzle exit of a liquid oxygen–kerosene engine emits high-speed, white, oxygen-rich gas at a temperature of approximately 600 K. The flame appearance and variation in the plume of a liquid rocket engine are highly dynamic. Cameras arranged around the test stand typically record 500 frames per second, generating over ten thousand high-speed images in a 30 s test.

Current methods rely on human observation to detect the presence and timing of flames in the plume, which has several of the following drawbacks: (1) Engine tests generate a large volume of plume image data, making manual interpretation time-consuming and prone to overlooking local details; (2) sensitivity to color varies among individuals, leading to inconsistent judgments about the presence of flames; (3) the human eye is not sensitive to absolute color values but only to changes in color, resulting in inconsistent evaluations of flames, even by the same observer under different conditions. Therefore, it is necessary to provide a flame classification model that is based on plume image features to automatically and rapidly classify flame and non-flame regions in the plume, enabling a precise determination of the fault onset time.

Currently, a substantial amount of research has been conducted on image-based fire detection. The key lies in separating the fire flames from environments with interfering objects. Common classification methods include Bayesian classifiers [1], support vector machines (SVM) [2,3,4], and Convolutional Neural Networks (CNN) [5,6]. Vision-based systems typically utilize three fire characteristics: color, motion, and geometric shape. Among these, color-based segmentation is suitable for flame extraction. In the visible spectrum, different color spaces are commonly used, including RGB [7,8,9], YCbCr [10], Lab* [11], HSV [12], or combinations of different color spaces [13,14,15].

Yamagishi et al. [16] proposed an effective method for flame detection by extracting spatiotemporal fluctuation data along the contour of the flame region based on color information. This method utilizes the HSV model and inputs the frequency component distribution of the spatiotemporal data into a neural network to achieve flame detection. Chen et al. [14] designed rules that were based on the RGB color model to distinguish flame pixels, with decisions primarily derived from the intensity and saturation of the R component for early fire alarms. However, this algorithm’s drawback is its low robustness to illumination changes when representing flames in the RGB color space. Horng et al. [17] extracted flame features using the HSI color model, roughly isolating regions with similar flame colors. They then employed image differencing methods and color mask techniques based on coloration to remove pseudo-flame sources such as objects with similar flame colors or flame reflection areas.

Marbach et al. [18] utilized the YUV color model to represent video data, where the temporal derivative of the luminance component Y was used to identify candidate fire pixels. The chrominance components U and V were then employed to classify these candidate pixels, proposing an image processing technique for real-time automatic fire detection in video images. To mitigate the effects of lighting variations, Celik et al. [19] proposed a normalized RGB approach. By conducting statistical analyses on the r-g, r-b, and g-b planes, they developed a general color model for flames using normalized RGB values. Additionally, Celik et al. [7] created a statistical model using 16,309,070 pixels gathered from 150 images of varying resolutions collected from the internet. They determined that each pixel in a fire should meet the following criteria: the red channel value must be greater than the mean red channel value of the entire image, the red channel value must be greater than the blue channel value, and the blue channel value must be greater than the green channel value.

Celik et al. [20] observed that flame pixels are more likely to have a Y component greater than the Cb component, and the larger the difference between Y and Cb, the more likely it is to be a flame pixel. They proposed a general color model for flame pixel classification based on rules, utilizing the YCbCr color space to more effectively separate luminance from chrominance, which proved superior to the RGB color space. Their method achieved higher detection rates, lower false alarm rates, and over 90% fire detection accuracy [10].

Chen et al. [21] employed the Lab*, YCbCr color spaces, and the *k*-means clustering algorithm to classify static flame images. This algorithm demonstrates high stability and flexibility for real-time and accurate forest fire detection. In another clustering-related study, Truong and Kin [22] proposed an effective method that first detects moving regions using an adaptive Gaussian Mixture Model (GMM) and then segments candidate fire regions based on flame color using the Fuzzy C-Means (FCM) algorithm. Vipin et al. [23] introduced an image processing technique using both RGB and YCbCr color spaces for forest fire detection, classifying fire pixels with a rule-based color model for real-time forest fire detection. Rossi and Akhloufi [24] used YUV and RGB color space information to segment fire regions, extracting salient points from a set of stereo images using clustering. They applied *k*-means clustering to the “V” channel to extract the most relevant fire regions, employing a two-stage segmentation technique for global fire region segmentation from unstructured scenes and multi-region subdivision for corresponding colored fire images.

Beyond color information, many studies also leverage dynamic, inter-frame, and motion cues to distinguish fire from fire-like objects, which provides a progressive logic for prior work. Toreyin et al. [25] used not only color and motion information but also detected flickering processes and spatial color variations in moving regions through a hidden Markov model. Ko et al. [8] identified candidate fire regions and then constructed a temporal fire model using wavelet coefficients, applying it to a binary support vector machine classifier with a radial basis function (RBF) kernel. Seebamrungsat et al. [26] utilized the HSV and YCbCr color models to separate orange, yellow, and high-brightness areas from the background. They analyzed and calculated fire growth using the frame difference method, achieving an overall accuracy rate of over 90% in their experiments.

Building on these ideas, subsequent studies have further combined motion cues with multi-space color modeling. Han et al. [27] proposed a fire detection method that fully utilizes the motion characteristics and color information of fire, first using GMM background subtraction for motion detection to extract moving objects from video streams. Subsequently, a combination of multi-color detection in RGB, HSI, and YUV color spaces was employed to identify potential fire regions. The flickering frequency is crucial for fire detection, as flames flicker at a specific frequency of approximately 10 Hz, which is independent of the burning materials and burners. Zhao et al. [28] utilized 11 static features, including color distribution, texture parameters, and shape roundness, as well as 27 dynamic features, such as changes in color, texture, roundness, area, and contour, to train a dynamic support vector machine (SVM) classifier for final decision-making. Triveni et al. [29] proposed a method for detecting flames based on extracting spatiotemporal fluctuation data on the flame region contours by using color information. They employed the HSV model and input the frequency component distribution of the spatiotemporal data into a neural network to achieve flame detection.

Related digital image analyses have also been used to monitor combustion states, reinforcing the role of inter-frame and spectral–color variations. Luo et al. [30] employed digital image processing techniques to extract flame image features, enabling continuous online monitoring of boiler combustion states. Huang et al. [31] conducted research on flame characteristics based on digital color analysis. They discovered that in the RGB and HSI (Hue, Saturation, Intensity) color models, the variations in the B and G color components are associated with CH* and CO emissions, respectively, reflecting the behavior of chemiluminescence under different combustion conditions.

Recent video fire detection research emphasizes tailoring the pipeline to the operating scenario to reduce false alarms while maintaining high recall. Representative systems integrate deep detectors with motion-based filtering and even vision–language verification to adapt to activity level and range, improving resilience in complex scenes [32,33]. In parallel, multilevel frameworks combine a modern detector with classical descriptors (e.g., texture or shape) to further stabilize precision under challenging backgrounds [34], and comparative studies quantify the accuracy–latency trade-offs across YOLO families for wildfire/smoke in ground and aerial imagery [35]. Closer to our design philosophy, hybrid schemes that couple deep backbones with lightweight, interpretable decision rules effectively suppress smoke-like phenomena and other distractors in resource-constrained deployments [36,37]. Comprehensive surveys also highlight that the method choice should reflect a scene’s range and background activity, and they identify evaluation gaps that are relevant to real deployments [38].

In this paper, we focus on liquid rocket engine exhaust plumes, which are highly transient, fast, and strongly turbulent. Under such conditions, inter-frame or motion cues are difficult to obtain and stabilize because of rapid brightness fluctuations, motion blur, and field test constraints. Motivated by these limitations, we concentrated on single-frame color information complemented by gradient features and designed the following detection pipeline. Section 2 introduces the RGB, YCbCr, and HSV color spaces to provide a multidimensional representation of color. Section 3 presents *k*-means segmentation in the YCbCr space to construct flame and non-flame sample sets. Section 4 builds a 10-dimensional feature vector for flame images and applies the Minimum Redundancy Maximum Relevance (mRMR) method to reduce dimensionality, improving computational efficiency and model performance. Section 5 details the SVM training parameters, the classification results, and an application to engine data. Section 6 discusses model limitations and future research directions, which is then followed by the conclusion.

## 2. Color Models

### 2.1. RGB Color Model

RGB encodes color with three additive channels—Red, Green, and Blue—and is widely used in imaging and displays. Its drawback is that brightness and chroma are entangled, so per-pixel RGB is sensitive to illumination and makes color attributes harder to determine robustly.

### 2.2. YCbCr Color Model

YCbCr separates luminance (Y) from chrominance (Cb/Cr)—blue-difference and red-difference components—which is commonly used in video and image compression. This decoupling aligns with human sensitivity to brightness, supports efficient coding, and improves robustness for color analysis and thresholding.

### 2.3. HSV Color Model

HSV describes color with hue (H), saturation (S), and value (V). Because it aligns more closely with human perception than RGB, it is convenient for color manipulation, segmentation, and filtering.

**Figure 1:**
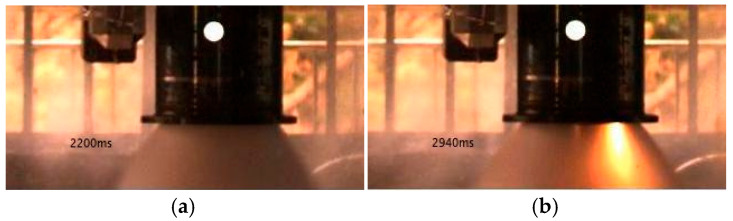
Representative test plumes. (**a**) **Normal plume:** oxygen-rich exhaust with low luminosity and no distinct bright combustion zones. (**b**) **Abnormal plume:** high-luminosity flame appears in the exhaust, indicating abnormal internal ablation within the engine.

Figure 1 shows images of the engine plume captured during the turbopump joint test of a liquid oxygen–kerosene engine. These images were obtained from real experimental data collected using a MEMRECAM ACS-1 M40 high-speed camera (nac Image Technology, Inc., Tokyo, Japan), which served as an optical sensor integrated into the test instrumentation system. The oxygen-rich gas expands at the nozzle exit, forming a trapezoidal plume. Under normal test conditions, the exit plume appears as shown in Figure 1a. However, if abnormal ablation occurs inside the engine during the test, bright flames may appear in the plume at certain moments, as illustrated in Figure 1b. Our subsequent image processing focuses specifically on the plume section at the engine exit.

**Figure 2:**
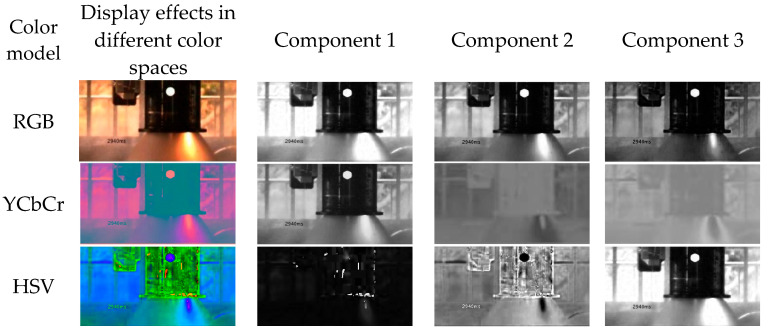
Flame images represented in different color spaces.

The most notable static features of flame images are color and brightness. The flame recognition algorithm primarily utilizes this color and brightness information in the video images. Flame color analysis determines whether a pixel is a flame pixel based on the relationship between the components in the color model. The flame region is significantly brighter than other regions, so the brightness component should be included in the color model. Figure 2 shows the plume images and their components represented in different color spaces. The experimental tests indicate that the best segmentation results are achieved when the images are processed in the YCbCr color space.

## 3. *k*-Means and YCbCr Color Segmentation

### 3.1. k-Means Clustering Analysis

The *k*-means clustering algorithm is an unsupervised machine learning technique that is used to partition a dataset into *k* distinct, non-overlapping clusters, maximizing intra-cluster similarity while minimizing inter-cluster similarity. The basic principles of the *k*-means algorithm are as follows:

1. Initialization: Randomly select *k* initial cluster centers (centroids).
2. Cluster Assignment: Assign each data point to the nearest centroid, forming *k* clusters.
3. Centroid Update: Recalculate the centroid of each cluster as the mean of all data points in the cluster.
4. Iteration: Repeat steps 2 and 3 until the centroids no longer change or a preset number of iterations is reached.

The *k*-means algorithm is straightforward, efficient, and easy to implement. However, it requires pre-specifying the number of clusters. The choice of initial centroids significantly impacts the final results, as different initial centroids can lead to different clustering outcomes. Moreso, *k*-means is often used in image processing and computer vision to segment images into multiple regions.

### 3.2. Flame Image Segmentation Based on k-Means Clustering

To construct a representative and informative dataset for model training, six images were deliberately selected from a single engine hot-fire test video that contained a known failure event. Only one fault event is available because faults are inherently rare in liquid-rocket hot-fire tests, and intentional fault induction is prohibited to protect the hardware. These frames were chosen based on the following criteria:

1. Visual diversity: The images vary in brightness, flame morphology, and background contrast, including high-luminosity flame cores, transitional flame edges, and dark non-flame regions.
2. Label clarity: All six images enable an intuitive visual interpretation of flame and non-flame areas, facilitating reliable ground-truth annotation and validation.

**Figure 3:**
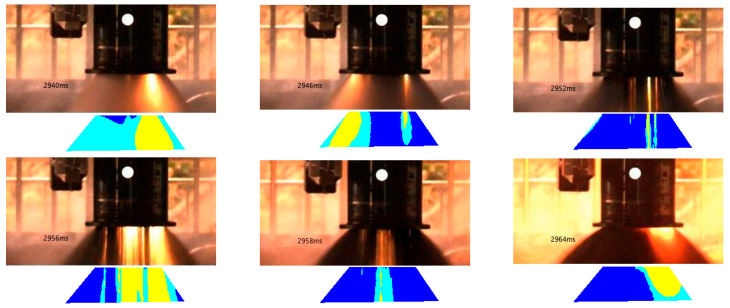
Segmentation results of plume images at different times based on *k*-means and YCbCr model.

Figure 3 presents the selected images and their segmentation results. To perform coarse segmentation, we applied the *k*-means clustering algorithm in the YCbCr color space. The number of clusters was set to *k* = 3, based on both visual feature separation and application-driven objectives. Specifically, the images consistently exhibit three distinguishable pixel zones:

1. Flame core region (labeled in yellow): This bright white or yellow area represents the high-temperature central part of the flame and is definitively classified as a flame region.
2. Flame periphery (labeled in cyan): This transitional area exhibits a dimmer glow surrounding the flame core and is also considered part of the flame.
3. Non-flame region (labeled in blue): Corresponding to the dark background of the plume, this area is identified as non-flame.

This three-class segmentation corresponds to meaningful physical and thermal distinctions in the plume structure and provides a robust basis for pixel-wise labeling into flame and non-flame classes.

We also experimented with other values of k (e.g., *k* = 2 and *k* = 4). When *k* = 2, the flame core and periphery were merged, resulting in under-segmentation that blurred the boundary between the flame and background. When *k* = 4, the segmentation became unstable and fragmented, increasing ambiguity in the labeling and reducing consistency. Therefore, setting *k* = 3 achieves a balance between interpretability, segmentation quality, and feature stability and provides a strong foundation for the subsequent feature extraction and classification process.

## 4. Feature Extraction and Dimensionality Reduction

### 4.1. Construction of Feature Vectors

**Figure 4:**
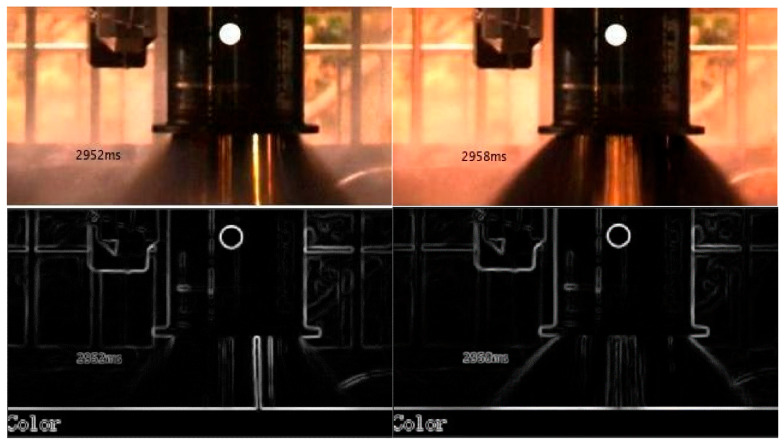
Plume images and their gradient representations at 2952 ms and 2958 ms.

The segmentation results of the six images in Figure 3 show a clear separation between the flame and non-flame regions, which is generally consistent with the visual structure of the plume. One exception is the image at 2952 ms, where two visible flame streaks were not captured by the clustering algorithm but can still be clearly distinguished by the human eye. Upon analysis, it was found that the *k*-means clustering algorithm cannot segment this region unless the number of clusters is increased to more than six (*k* = 6). However, this would significantly degrade the segmentation results in other areas. Considering that the human eye can distinguish this region using the brightness difference between the pixels in this region and the surrounding pixels, another image feature was used to describe the flame feature, namely, the magnitude of the image gradient. Figure 4 shows the gradient image of this image, where the flame region can be clearly identified from the gradient image.

To describe the size and shape of the flame, this study selected the basic color components from different color space models and gradient magnitudes as features. Ten static flame features were extracted, resulting in a 10-dimensional feature vector consisting of R, G, B, H, S, V, Y, Cb, Cr, and M (magnitude of the gradient). Specifically, R, G, and B refer to the red, green, and blue components of the RGB color model. H, S, and V are the color features in the HSV color space model, while Y, Cb, and Cr are the color features in the YCbCr color space model. M represents the magnitude of the image gradient at each pixel.

### 4.2. Feature Vector Dimensionality Reduction

**Figure 5:**
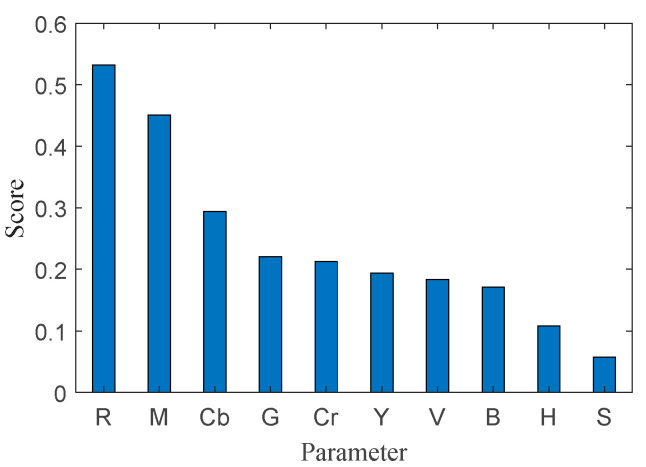
Feature ranking based on mRMR.

Using mRMR to rank the 10-dimensional candidate features, the scores are shown in Figure 5. The importance order is R, M, Cb, G, Cr, Y, V, B, H, and S. Based on repeated experiments, we selected the top five features—R, M, Cb, G, and Cr—for SVM training. The top-ranked subset reflects plume physics and imaging constraints. R and Cr capture the red-dominant core while decoupling luminance; Cb and G provide complementary contrast for peripheral rims and wall background; and the gradient magnitude M delineates thin, turbulent boundaries with reduced sensitivity to illumination swings. Together, these cues are mutually complementary and minimally redundant, which explains their selection by mRMR. This subset retains both color features (R, Cb, G, and Cr) and the gradient magnitude (M), thereby preserving the edge cues while reducing redundancy. These features constitute the final subset used for SVM training.

## 5. Model Training

### 5.1. Gaussian SVM Model

Support vector machine (SVM) is a supervised learning model that is based on the principle of structural risk minimization. SVM demonstrates excellent performance in solving small sample, nonlinear, and high-dimensional pattern classification problems. By optimizing the objective function, SVM can find the global optimal solution to the classification problem, rather than a local optimal solution, effectively avoiding the issue of local minima that traditional neural networks often encounter. SVM enhances the model’s generalization ability by maximizing the classification margin, performing well even on high-dimensional or small sample datasets. For cases that are not linearly separable, SVM introduces kernel functions to map samples to a high-dimensional space where linear classification can be achieved. The choice of the kernel function is critical. The experimental results show that selecting the Gaussian kernel function yields the best performance. The definition of the Gaussian kernel function is given by Equation (1):

(1) $$K(x,y)=\exp(-\gamma {\left\|x-y\right\|}^{2})$$

where γ is the kernel scale parameter, which controls the width of the Gaussian kernel.

The goal of SVM is to find an optimal hyperplane to classify samples. For nonlinear separable data, SVM uses the Gaussian kernel function to map the data into a higher-dimensional space. In this high-dimensional space, the optimization problem can be formulated as:

(2) $$\underset{\omega ,b,\xi }{\min}\frac{1}{2}{\left\|\omega \right\|}^{2}+C\sum_{i=1}^{n}\xi_{i}$$

where ω is the normal vector of the hyperplane; *b* is the bias term; $\xi_{i}$ is the slack variable, representing the degree of misclassification for the *i*-th sample; and *C* is the penalty factor, which controls the trade-off between maximizing the margin and minimizing the classification error.

The constraints are:

(3) $$y_{i}(\omega^{T}\phi (x_{i})+b)\geq 1-\xi_{i},\xi_{i}\geq 0$$

where $\phi (x_{i})$ is the mapping of sample $x_{i}$ into the higher-dimensional space using the kernel function.

To solve the above optimization problem, we introduce Lagrange multipliers. The dual form of the optimization problem is:

(4) $$\underset{\alpha }{\max}\sum_{i=1}^{n}\alpha_{i}-\frac{1}{2}\sum_{i=1}^{n}\sum_{j=1}^{n}\alpha_{i}\alpha_{j}y_{i}y_{j}K(x_{i},x_{j})$$

where $\alpha_{i}$ are the Lagrange multipliers.

The constraints are:

(5) $$\sum_{i=1}^{n}\alpha_{i}y_{i}=0,0\leq \alpha_{i}\leq C$$

After training the SVM model, the decision function is:

(6) $$f(x)=\sum_{i=1}^{n}\alpha_{i}y_{i}K(x_{i},x)+b$$

where *b* is the bias term, calculated as follows:

(7) $$b=y_{i}-\sum_{j=1}^{n}\alpha_{j}y_{j}K(x_{j},x_{i})$$

The class of a new sample *x* is determined by the sign of the decision function f(x):

(8) class=sign(f(x))

### 5.2. Model Training Settings

In addition to the six frames of clustered images containing flame and non-flame pixels, we included two additional images with no flames. This resulted in a total of 22,190 positive samples (flame pixels) and 35,613 negative samples (non-flame pixels) for the training dataset. The positive samples encompass flames of various colors, shapes, and intensities. The five parameters $sv=\left\{R,M,Cb,G,Cr\right\}$ extracted for each pixel, as discussed in the previous section, were used as the input feature vectors for training the SVM classifier. The input feature vectors were standardized to ensure comparability across different features. Standardization was achieved by subtracting the mean and dividing by the standard deviation of each feature.

In SVM, the penalty factor C and the kernel parameter γ for the Gaussian kernel function are crucial parameters that significantly impact model performance. The penalty factor C controls the penalty for misclassified samples. A high C value imposes a greater penalty on misclassifications, leading the model to minimize misclassifications but possibly at the expense of a smaller margin, which can result in overfitting. The kernel parameter γ determines the “width” of the Gaussian kernel, i.e., the influence range of each training sample. A high γ value indicates a smaller influence range, making the decision boundary more complex and potentially leading to overfitting.

### 5.3. Model Training and Validation

Cross-validation is a technique used to evaluate the generalization ability of a model by dividing the dataset into multiple subsets (typically *k*-folds) for repeated training and testing. This approach effectively mitigates overfitting by averaging the accuracy of each test, ensuring that all samples are used for both training and testing, thereby enhancing the reliability of the evaluation results. In this study, 5-fold cross-validation was employed to validate the model’s accuracy, with 80% of the data used for training and 20% for validation, repeated five times.

For this study, the penalty parameter C was set to 1, a relatively small value, indicating a lower penalty for misclassifications. This choice was influenced by the fact that flame pixels were identified through clustering analysis combined with subjective judgment, which may not be entirely accurate. The γ value was adjusted to 0.56 through experimentation to achieve optimal results. The final model achieved an accuracy of 97.6%, where accuracy is defined as the proportion of correctly classified samples out of the total samples. The total cost was calculated based on the cost matrix of misclassifications.

### 5.4. Confusion Matrix and Classification Performance Metrics

The confusion matrix is a table used to evaluate the performance of a classification model. It displays the cross-tabulation of actual and predicted classes, with each row representing the actual class and each column representing the predicted class. The diagonal elements indicate the number of correctly classified samples, while the off-diagonal elements indicate the number of misclassified samples. The confusion matrix in this study is a 2 × 2 matrix, as shown in Table 1.

Based on the confusion matrix, the true positive rate (TPR) of the classification model is 98.56%, and the false positive rate (FPR) is 3.99%.

**Figure 6:**
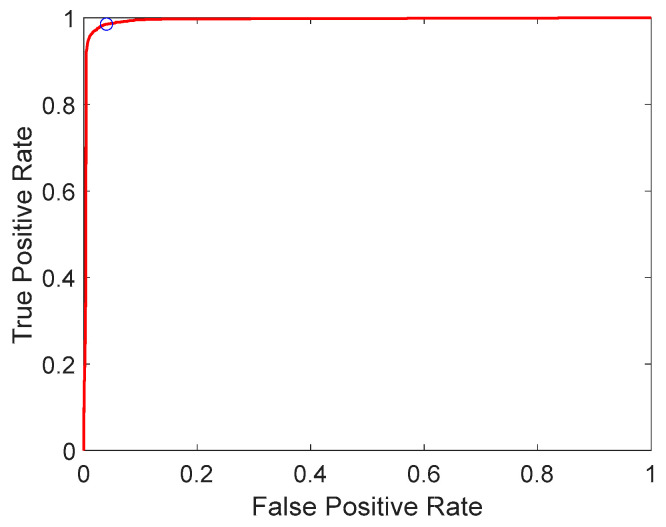
ROC curve.

The Receiver Operating Characteristic (ROC) curve is a crucial tool for evaluating the performance of binary classification models. It illustrates the relationship between the TPR and FPR across different classification thresholds. By plotting the TPR against the FPR at various threshold settings, the ROC curve provides a comprehensive view of classifier performance. As shown in Figure 6, the ROC curve for the image dataset demonstrates the model’s behavior as the threshold *τ* varies from −6.3365 to 6.4219. By examining the ROC curve, we can select the optimal threshold that balances the TPR and FPR, thereby enhancing the model’s classification performance. Each point on the ROC curve corresponds to three associated values: the TPR, FPR, and threshold *τ*. Given that the flame recognition model in this study must not miss any potential flames, it is crucial to choose a threshold *τ* that results in a high TPR. In our case, we selected a threshold that yields a TPR of 98.56%, with a FPR of 3.99%, which is within an acceptable range; the circle in Figure 6 highlights the final operating point (selected threshold). The overall performance of the classification model can be quantified by calculating the area under the ROC curve (AUC). The AUC ranges from 0 to 1, with higher values indicating better classification performance. In our experiments, the AUC for the ROC curve was 0.99, indicating that the model exhibits excellent classification capability. By using the ROC curve and AUC, we can rigorously evaluate and optimize the performance of our flame recognition model, ensuring high accuracy and reliability in identifying flames.

### 5.5. Visualization and Comparison of Classification Results

**Figure 7:**
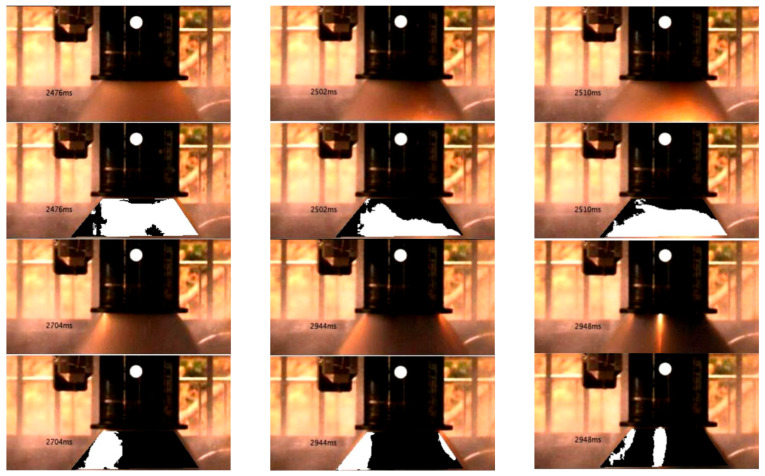
Flame recognition results at different time points based on the established SVM model.

To further evaluate the performance of the trained SVM model, we applied it to image sequences captured at different moments during the test run. The results are illustrated in Figure 7, where the detected flame regions are marked in white, and the non-flame regions are marked in black.

The visualization demonstrates that the model can accurately identify the flame regions. The classified flame areas and shapes exhibit a high degree of agreement with those determined by human visual interpretation. This consistency underscores the model’s reliability and effectiveness in flame detection applications.

By using the established model, the exact timing of flame onset can be intuitively determined, providing support for fault analysis. This model eliminates discrepancies in flame onset timing judgments among different researchers and has received unanimous recognition from engine designers.

### 5.6. Application of the Model in Hot-Fire Test Runs

**Figure 8:**
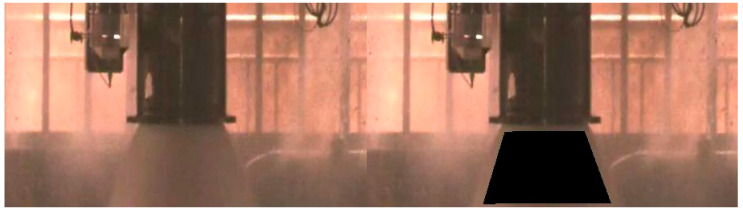
Using the established model to determine the absence of flames in plume images from other test runs.

The established SVM model has played a critical role in multiple test runs, serving as a vital tool for identifying the presence of flames in the exhaust plume and predicting potential abnormal ablation within the engine. By applying this model, we were able to determine that no flames appeared at any point in the exhaust plume during subsequent engine test runs, as illustrated in Figure 8.

This model has become a crucial decision-making aid in determining whether the engine requires disassembly for inspection or if it is safe to proceed with further testing. The consistent and reliable performance of the SVM model in detecting flames ensures its utility in maintaining engine safety and optimizing the testing process.

## 6. Discussion

In this study, we aimed to enhance intelligent sensing and monitoring capabilities during engine development by developing and validating a machine learning–based flame detection model using data that were acquired from optical image sensors. Specifically, we constructed a Gaussian SVM model that leverages both color and gradient features. By analyzing exhaust plume images collected in real experimental environments during actual engine test runs, the model successfully identified flame regions and predicted potential abnormal ablation inside the engine. Notably, it detected flame occurrences more than 0.5 s earlier than current fault detection systems.

In practical applications, this SVM model can be integrated into engine fault detection systems to monitor plume images in real time. Once abnormal flames are detected, the system can immediately issue alerts, prompting test personnel to conduct further inspections and thereby enhancing test safety.

Nevertheless, this study has several limitations. First, the dataset is derived from a single fault event and is therefore limited in scale, as engine test faults are inherently costly and resource-intensive. Second, due to background differences, a model trained on one test stand is difficult to generalize to other test stands. Finally, since the model is designed specifically for flame detection, it is applicable only to turbopump integration tests. For full engine hot-fire tests, in which the entire plume consists of flames, this method would inevitably fail.

Future research directions include the following: (1) developing more sophisticated feature extraction methods to further improve flame detection accuracy; (2) integrating more sensor data, such as infrared temperature and pressure data, to enhance the model’s diagnostic capabilities; and (3) conducting more tests under real experimental conditions to verify the model’s practical utility. Moreover, this work has initiated the development of an engine fault diagnosis framework based on plume spectroscopy, which can be applied to full engine hot-fire tests.

## 7. Conclusions

In this study, we proposed and validated a Gaussian SVM-based flame detection model utilizing color and gradient features that were extracted from high-speed plume images that were captured by optical sensors during liquid rocket engine tests. The model effectively distinguishes flame from non-flame pixels and achieved a classification accuracy of 97.6% using a limited training dataset. Its consistent performance and early detection capability demonstrate strong potential for enhancing intelligent sensing and fault monitoring in turbopump joint tests, particularly in identifying signs of abnormal ablation within the engine.

Despite its promising results, the model was developed based on data from a single fault event, limiting its generalizability. To improve robustness and adaptability, future research will focus on expanding the dataset, exploring more advanced feature extraction techniques, and incorporating additional sensing modalities such as infrared and pressure data. Furthermore, efforts will be made to improve real-time detection performance, paving the way for the integration of such models into closed-loop engine health monitoring systems.

## Figures and Tables

**Table 1:** Confusion matrix.

Actual\Predicted	Positive (Predicted)	Negative (Predicted)
Positive (Actual)	35,102	511
Negative (Actual)	885	21,305

## Data Availability

The raw data supporting the conclusions of this article will be made available by the authors on request.

## Abbreviations

The following abbreviations are used in this manuscript:

AUC	Area Under the (ROC) Curve
CNN	Convolutional Neural Network
FCM	Fuzzy C-Means (clustering)
FPR/TPR	False Positive Rate/True Positive Rate
GMM	Gaussian Mixture Model
mRMR	Maximum Relevance Minimum Redundancy
RBF	Radial Basis Function (Gaussian kernel)
ROC	Receiver Operating Characteristic
SVM	Support Vector Machine
